# Predictive Factors for Neutralizing Antibody Levels Nine Months after Full Vaccination with BNT162b2: Results of a Machine Learning Analysis

**DOI:** 10.3390/biomedicines10020204

**Published:** 2022-01-18

**Authors:** Dimitris Papadopoulos, Ioannis Ntanasis-Stathopoulos, Maria Gavriatopoulou, Zoi Evangelakou, Panagiotis Malandrakis, Maria S. Manola, Despoina D. Gianniou, Efstathios Kastritis, Ioannis P. Trougakos, Meletios A. Dimopoulos, Vangelis Karalis, Evangelos Terpos

**Affiliations:** 1 Section of Pharmaceutical Technology, Department of Pharmacy, School of Health Sciences, National and Kapodistrian University of Athens, 15784 Athens, Greece; d.papadopoulos89@gmail.com; 2 Department of Clinical Therapeutics, School of Medicine, National and Kapodistrian University of Athens, 11528 Athens, Greece; johnntanasis@med.uoa.gr (I.N.-S.); mgavria@med.uoa.gr (M.G.); panosmalan@med.uoa.gr (P.M.); ekastritis@med.uoa.gr (E.K.); mdimop@med.uoa.gr (M.A.D.); 3 Department of Cell Biology and Biophysics, Faculty of Biology, National and Kapodistrian University of Athens, 15784 Athens, Greece; zoievag@biol.uoa.gr (Z.E.); mmanola@biol.uoa.gr (M.S.M.); gndespoina@biol.uoa.gr (D.D.G.); itrougakos@biol.uoa.gr (I.P.T.)

**Keywords:** SARS-CoV-2, COVID-19, neutralizing antibodies, machine learning, principal component analysis, factor analysis of mixed data, k-means clustering, random forest

## Abstract

Vaccination against SARS-CoV-2 with BNT162b2 mRNA vaccine plays a critical role in COVID-19 prevention. Although BNT162b2 is highly effective against COVID-19, a time-dependent decrease in neutralizing antibodies (NAbs) is observed. The aim of this study was to identify the individual features that may predict NAbs levels after vaccination. Machine learning techniques were applied to data from 302 subjects. Principal component analysis (PCA), factor analysis of mixed data (FAMD), k-means clustering, and random forest were used. PCA and FAMD showed that younger subjects had higher levels of neutralizing antibodies than older subjects. The effect of age is strongest near the vaccination date and appears to decrease with time. Obesity was associated with lower antibody response. Gender had no effect on NAbs at nine months, but there was a modest association at earlier time points. Participants with autoimmune disease had lower inhibitory levels than participants without autoimmune disease. K-Means clustering showed the natural grouping of subjects into five categories in which the characteristics of some individuals predominated. Random forest allowed the characteristics to be ordered by importance. Older age, higher body mass index, and the presence of autoimmune diseases had negative effects on the development of NAbs against SARS-CoV-2, nine months after full vaccination.

## 1. Introduction

The novel Severe Acute Respiratory Syndrome Coronavirus 2 (SARS-CoV-2) has caused a worldwide epidemic and has become a serious global public health threat [1,2]. The coronavirus genome encodes four different major structural proteins: spike, envelope, membrane, and nucleocapsid. ACE2 receptors are typically found on epithelial cells of the oral mucosa and alveolar lung cells II but can also be found in other human organs [3]. The virus enters the body via the viral S protein and binds to ACE2 receptors [3]. COVID-19 is a systemic disease that causes both short- and long-term symptoms [4,5,6]. According to the literature, the vast majority of affected individuals have mild to moderate symptoms, with 5% to 10% having a severe or life-threatening course of disease. Worldwide, the development of effective and safe vaccines and drugs, as well as new diagnostics and therapies, is a top priority.

Vaccination against SARS-CoV-2 is ongoing worldwide, with BNT162b2 mRNA vaccine playing a critical role in most countries [7,8,9]. After immunization, healthy individuals exhibit significant levels of IgG antibodies and neutralizing antibodies (NAbs) directed against the spike receptor binding domain of SARS-CoV-2, as well as a protracted B-cell response in the germinal center [10,11]. It is worth noting that NAbs content has been associated with clinically significant immune protection against COVID-19 [12,13].

Although BNT162b2 is highly effective against COVID-19, a time-dependent decrease in antibody levels against SARS-CoV-2 was observed in vaccinated individuals [14,15,16,17,18,19]. Even one month after the second BNT162b2 injection, there was a modest decline in antibody titers, and the time since the second vaccine dose was associated with decreased neutralizing antibody activity against SARS-CoV-2 variants and attenuated protection against COVID-19 [14,15,16,17,18]. An important question to answer is the identification of characteristics of individuals that may act as predictive factors for neutralizing antibody levels long after vaccination.

A useful tool to answer this question is the application of machine learning techniques (ML), a subfield of artificial intelligence [20,21]. The goal of ML is generally to understand the structure of data and to fit that data into models that can be understood and used by humans. ML techniques allow data to be trained and then statistical analysis applied to produce values that fall within a certain range [20,21]. Thus, ML enables the development of models from sample data to automate decision-making processes based on data inputs. Based on how the system learns or receives feedback on what it learns, ML tasks are generally classified into broad groups. The two most commonly used ML methods are “supervised learning,” in which algorithms are trained based on labeled input and output data, and “unsupervised learning,” in which the algorithm is not provided labeled data to discover structure in the input data [20,21].

Most studies investigating the immune response after vaccination rely on the application of classical statistical approaches, neglecting the strong analytical power of machine learning methods. The aim of this study was to apply four popular ML methods to identify factors responsible for NAbs levels nine months after vaccination with BNT162b2. Data from an ongoing clinical trial of 302 subjects were used to unveil hidden relationships between subject characteristics and NAbs that are difficult or impossible to find using classical statistical approaches. To take advantage of both supervised and unsupervised methods, a combination of both methods was used in this analysis. In addition, three unsupervised methods were used, which complement each other.

## 2. Materials and Methods

### 2.1. Clinical Study

The clinical trial was conducted at Alexandra General Hospital in Athens, Greece (NCT04743388), after approval by the Ethics Committee of the institution. The entire clinical setting was conducted in accordance with the Declaration of Helsinki and the International Conference on Harmonization of Good Clinical Practice. All study participants gave informed consent before participating in the study. Primary inclusion criteria included the ability to sign informed consent, vaccination with BNT162b2 mRNA vaccine, and age older than 18 years. Individuals who had active malignant disease or end-stage renal disease or who were receiving immunosuppressive medications were excluded from participation in the study. Data confidentiality was ensured in accordance with the General Data Protection Regulation.

### 2.2. Antibodies Measurement

The schedule for blood collection in this clinical trial was as follows: on day 1 (D1) before the first vaccination, one week later (i.e., day 8, D8), three weeks later (i.e., day 22, the day of the second vaccination), and at the following time points after the second injection: two weeks, one month (M1), three months (M3), six months (M6), and nine months (M9).

Within 4 h of collection, blood was drawn and serum was extracted. The serum was then frozen at −80 °C until the day of measurement. NAbs against SARS-CoV-2 were identified using an FDA-approved technique. In this study, the cPassTM SARS-CoV-2 NAbs Detection Kit from GenScript (GenScript, Inc.; Piscataway, NJ, USA) was used, which allows indirect detection of SARS-CoV-2 NAbs in blood. The Elecsys Anti-SARS-CoV-2 S assay, which detects response to previous infection or immunization, was used to determine anti-S-RBD IgG antibodies (Roche Diagnostics GmbH, Mannheim, Germany).

### 2.3. Data Collection

At the beginning of the study, patient demographics (e.g., age, sex), medical history, and medication were obtained in an interview. Body mass index (BMI) was calculated based on the individual’s weight and height.

Many of the participants had dyslipidemia as well as cardiovascular problems, diabetes, and autoimmune diseases (e.g., psoriasis, atopic dermatitis). Allergies (e.g., bronchial asthma, previous drug reactions), thyroid problems (e.g., Hashimoto’s disease), psychological problems (e.g., depression, migraine), and gastrointestinal disorders (e.g., gastroesophageal reflux disease) were also noted in the patients’ medical history. Due to the small sample size, the diseases in this study were divided into two categories: those with autoimmune diseases and those without.

### 2.4. Machine Learning

ML algorithms were used to analyze the percent inhibition thresholds (i.e., NAbs) with respect to all data from the above subjects. In ML, tasks are divided into two categories: supervised learning and unsupervised learning. In supervised learning, the computer is fed examples of inputs labeled with expected outputs. The goal of this method is for the algorithm to “learn” by comparing its real outputs to the “learned” outputs to detect errors and adjust the model accordingly. The supervised learning algorithm used in this study was Random Forest.

Since the data is not labeled in unsupervised learning, it is left to the learning algorithm to discover commonalities between the input data. Unsupervised learning can have the simple purpose of detecting hidden patterns in a data set, but it can also have the goal of feature learning, which allows the computational engine to automatically discover the representations needed to classify the raw data. In this work, k-means cluster analysis and two-dimension reduction approaches (principal component analysis and factor analysis of mixed data) were used.

Before applying the ML approaches, the data were tested for multi-collinearity, i.e., the characteristics of the independent participants were examined to see if they correlated with each other. Multi-collinearity has a significant impact on the variance associated with the problem and may also affect model interpretation by undermining the statistical significance of the independent variables. For all pairs of variables, Spearman’s correlation coefficient (i.e., r) was used to estimate correlation.

### 2.5. Principal Component Analysis

Principal component analysis (PCA) is a popular approach to transform a high-dimensional set of features into a low-dimensional set of features [22]. The goal of PCA is to find the lowest dimensional representation of the data while capturing as much information/variance as possible. PCA transforms the original space formed from the original dataset into a new space that is a linear combination of the dimensions of the dataset. Each additional dimension that is developed is called a principal component (PC). The new coordinates of the data are called “scores”.

PCA is a technique for reducing the dimensions of a data matrix in an attempt to capture as much variability as possible. Each PC explains a portion of the variance in the original data set. The direction of the first principal component is the direction in which the data varies the most. The contribution of each original dimension to the new dimension is quantified by various factors called “loadings.” Each principal component is the normalized linear combination of the original features, where normalized means that the squared sum of the loadings of each principal component equals one. The closer the loading value is to +1 (or 1), the more positive (or negative) the contribution of that feature to PC.

The best way to visualize the loadings and scores together is to use the “biplot”. The biplot is a two-dimensional scatter plot where the two axes represent the two most important PCs in terms of variance explained. The data points are plotted in this two-dimensional coordinate system, using the scores as coordinates, and the loadings of the first two PCs of each trait are plotted over the data points.

Scree plots were used to determine the least number of principal components needed to accurately represent the original data. The main purpose of a scree plot is to show the results of the component analysis and to locate the apparent change in slope (elbow). In a scree plot, the eigenvalue is plotted against the principal components. The eigenvalue of a component divided by the sum of the eigenvalues is the proportion of the variance explained by that component. The first component usually explains a large portion of the variability, the next components explain a moderate portion, and the last components explain only a small portion of the total variability.

### 2.6. Factor Analysis of Mixed Data

While PCA works well on continuous data, it is ineffective on categorical data because the resulting PCs aim to maximize the variability of the underlying data set in the new, transformed, lower-dimensional space. This is because categorical features require “one-hot coding” that transforms them into binary features. The concept of variability is consistent with binary features, as is the use of PCA.

Factor analysis of mixed data (FAMD) is a technique that combines PCA with multiple correspondence analysis to analyze numerical and categorical variables [22]. By considering both continuous and categorical data, FAMD also leads to a low-dimensional space. The results can be summarized in a biplot as in PCA, as mentioned above. In addition, as with PCA, scree plots were created to determine the required number of principal components.

### 2.7. K-Means Cluster Analysis

When the data is unlabeled, another popular unsupervised learning technique is k-means cluster analysis, which creates groups (clusters) of variables from the original dataset [22]. K-means partitions a *p*-dimensional space into *k* groups (where p is the number of variables in the dataset). Each of the *k* clusters is defined by a centroid, which, as the name implies, is located at the center of the cluster. Each point in the dataset is assigned to the cluster with the closest centroid. A meaningful interpretation of the clusters is possible by looking at the coordinates of the centroid.

To determine the optimal number of clusters, the “elbow” approach (scree plot) was used. For different values of *k*, the sum of squared distances between each point and the centroid in a cluster (Within-Cluster Sum of Squares, WCSS) is determined. A line plot is then created with the squared distances on the y-axis and the different *k* values on the x-axis. The ideal k is the point at which the line plot forms an “elbow” (i.e., an angle).

In addition, the silhouette score and Davies-Bouldin index were calculated to determine the optimal number of clusters. The silhouette score is used to evaluate the quality of clusters created using clustering methods, i.e., to assess how well samples cluster with other samples that are similar to each other. The silhouette score is created for each sample from each cluster and ranges from −1 to +1, with a high number (close to 1) indicating that the object matches its own cluster well. A value less than 0 indicates that the data from the clusters may not be correct. Negative values often indicate that a sample has been assigned to the wrong cluster. Similarly, the Davies-Bouldin index is a validation metric used to determine the best number of clusters. The minimum value is zero, with lower values indicating better clustering.

### 2.8. Random Forest

In the context of machine learning, bagging is a technique in which numerous “copies” of the training data are made (each “copy” being slightly different from the others). Then a weak learner, such as a “decision tree”, is applied to each copy. In this way, many weak models are generated, which are then integrated. Random forest is a bagging approach of supervised learning, where a large number of decorrelated trees are created and then combined by averaging to obtain a more accurate and stable prediction of the target variable [22]. To make the model more robust, it is common to divide the original dataset into two sections, “train” and “test”. The training dataset is used to train the model and the test dataset is used to evaluate the performance of the model.

In a classification problem (i.e., when the target variable is categorical), the confusion matrix can be used to evaluate the performance of a model. The confusion matrix is an M × M matrix, where M is the number of target classes of the target variable. The matrix contrasts the true and expected classes. This provides a comprehensive view of the overall performance of the categorization model and the types of errors it makes. The accuracy and misclassification of the predictions can be calculated to reflect the performance of the classification. By examining the feature importance, it is also possible to see the contribution of each feature to the prediction of the target variable when using random forest.

## 3. Results

### 3.1. Demographics

BNT162b2 mRNA vaccine was administered in two doses to the 302 study participants. Demographic data and information on concomitant diseases/medications were available for all these subjects in addition to NAbs values.

Neutralizing antibody activity was determined at days one, eight, and twenty-two (prior to the second immunization), and one, three, six, and nine months thereafter. Table 1 summarizes the demographic information of the study participants. The mean age was 48 years (range, 49 years), females accounted for 65.4%, and males accounted for the remainder (34.6%). The median BMI was 24.8 (range = 27), and women had lower median BMI values than men (23.3 vs. 26.8).

### 3.2. Neutralizing Antibody Levels

**Figure 1:**
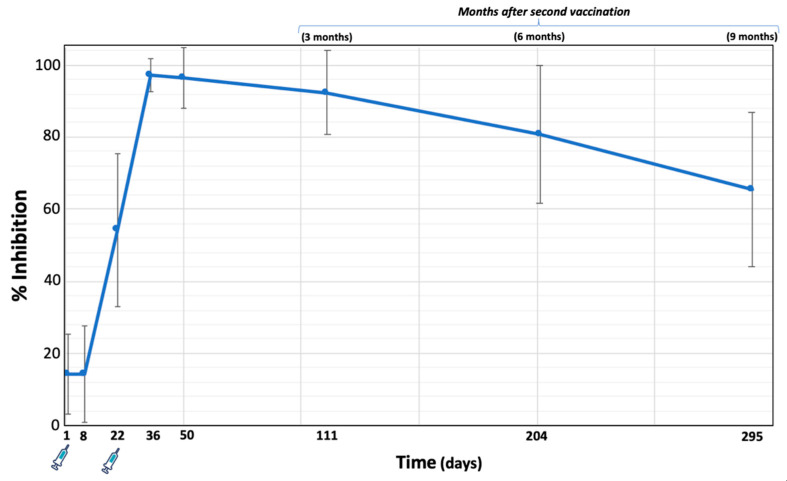
Mean percent inhibition (±standard deviation) of SARS-CoV-2 binding to the human host receptor angiotensin converting enzyme-2 after vaccination with the BNT162b2 mRNA vaccine in 302 subjects. Neutralizing antibody levels were measured on day 1 (first vaccination day), 8, 22 (second vaccination day), two weeks later, and one month, three months, six months, and nine months (i.e., 295 days from the initiation of the study) after the second vaccination.

Figure 1 shows the mean NAbs values (±standard deviation) from the day of the first vaccination to nine months after the second vaccination with the BNT162b2 mRNA vaccine.

At D1 and D8, median NAbs titers were low (14.23% and 14.28%, respectively), indicating that neutralizing antibodies are not formed within the first week after vaccination. Three weeks later, i.e., on the day of the second vaccination, the median value of inhibition was 54.19%, while two weeks later the maximum value is reached (97.24%). From this point on, the inhibition decreases continuously and nine months after the second vaccination the median value of NAbs is 65.43%.

### 3.3. Principal Component Analysis and K-Means Clustering

**Figure 2:**
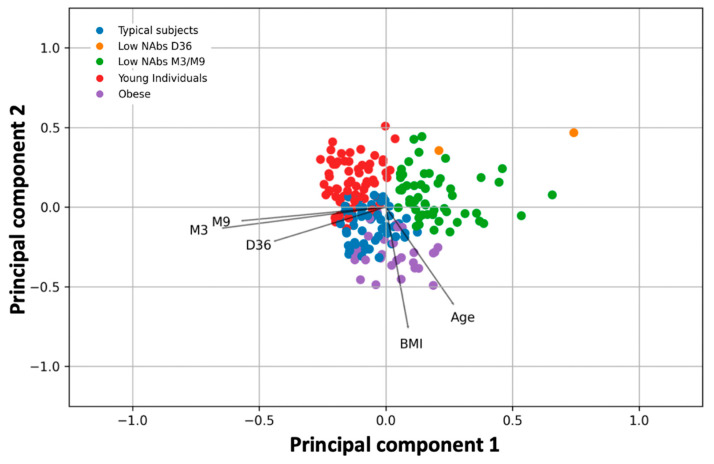
Principal component analysis of the features used in the study. The color of the dots is from the k-means cluster analysis, which divides the subjects into five groups, each with one distinguishing feature. The coordinates of the two-dimensional plot refer to the “scores” of the variables in the dimensionality-reduced space (two dimensions (principal components) are shown in the plot). *Key*: D36, neutralizing antibody levels two weeks after second vaccination; M3 neutralizing antibody levels three months after second vaccination; M9, neutralizing antibody levels nine months after second vaccination; BMI, body mass index.

**Figure A1:**
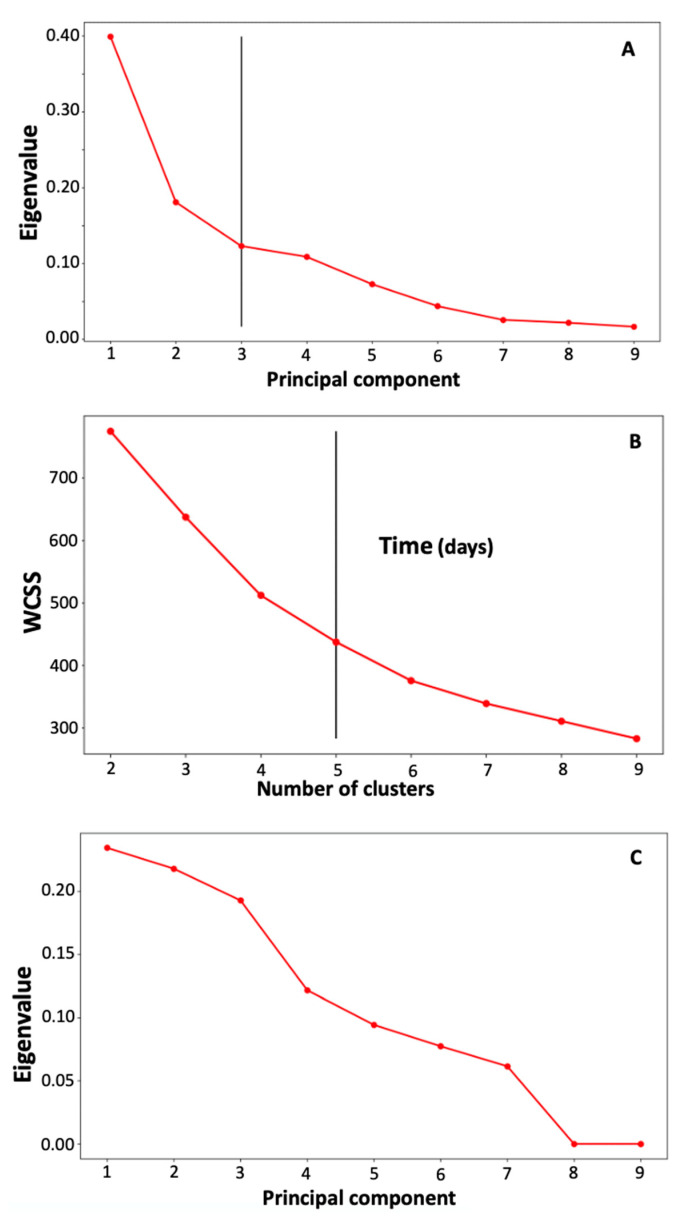
Scree (elbow) plots to determine the number of principal components or clusters in principal component analysis (**A**), k-means clustering (**B**), and factor analysis of mixed data (**C**). The *x*-axis refers to the number of principal components or clusters, while the *y*-axis corresponds to eigenvalues (for **A**,**C**) and the Within-Cluster Sum of Square (WCSS) (for plot **B**).

**Figure A2:**
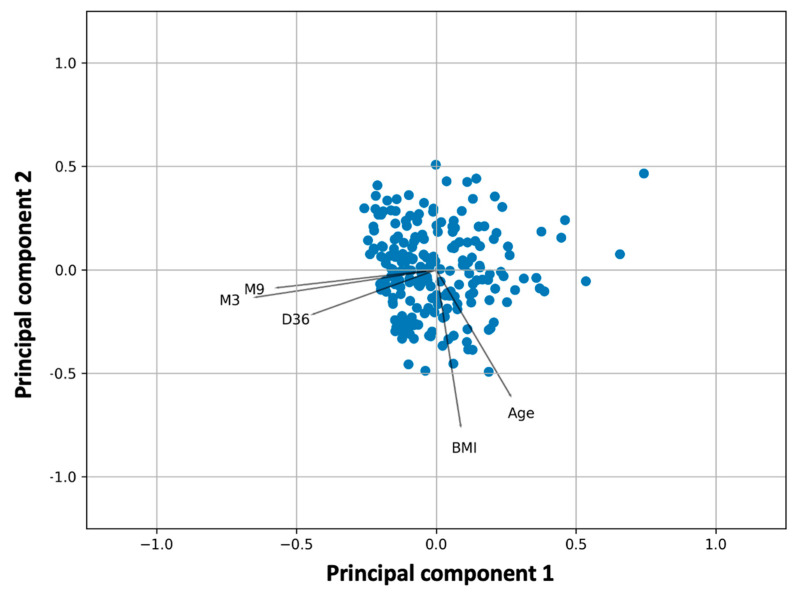
Principal component analysis of percent inhibition of SARS-CoV-2 binding at day 36 and at the third and ninth months after vaccination. The coordinates of the two-dimensional plot refer to the “scores” of the variables in the dimensionality reduced space (two principal components are shown). *Key*: D36, neutralizing antibody levels two weeks after second vaccination; M3 neutralizing antibody levels three months after second vaccination; M9, neutralizing antibody levels nine months after second vaccination; BMI, body mass index.

**Figure A3:**
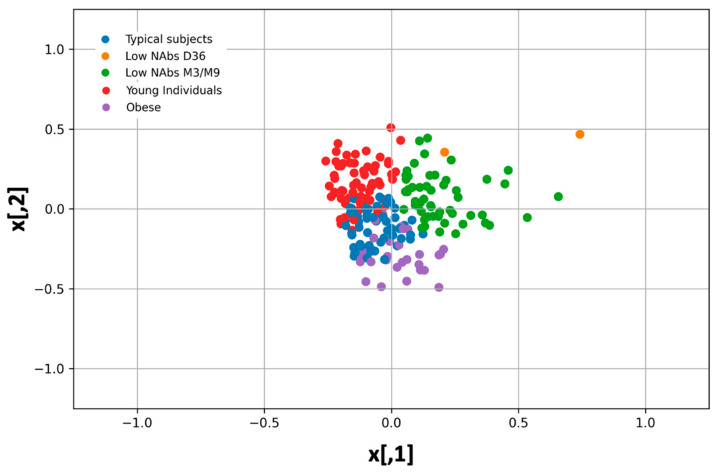
K-means cluster analysis plot of all features used in the study (neutralizing antibody levels and demographics). The two axes are labelled as x[,1] and x[,2]. In each cluster, shown with different color, a feature predominates.

To extract the participants’ information and examine its relationship with NAbs levels, principal component analysis was performed. Figure 2 shows the results of the PCA analysis superimposed on those of the k-means cluster analysis. The observations (study participants) are shown as dots in the plane formed by the two principal components, whereas the lines represent the vectors of the variables, namely the NAbs levels at D36, M3, and M9, and BMI and age. The different color of the points refers to the grouping from the k-means cluster analysis. Overlaying PCA and k-means clustering plots allows simultaneous evaluation and comparison of the results from the two methods. Scree plots were created to select the optimal number of principal components (Figure A1A) and clusters (Figure A1B). Separate plots of PCA and k-means clustering can be found in Figure A2 and Figure A3 in the Appendix A.

The first two principal components explain 63.4% of the total variability (39.8% and 23.6% for the first and second components, respectively). Visual inspection of Figure 1 shows that M3 and M9 are adjacent to each other on the left side of the plot near the first principal component, while D36 is also close to them, indicating their strong relationship. With respect to the first principal component, the loadings of D36, M3, and M9 are −0.44, −0.57, and −0.64, respectively (Table 2). This means that someone who has high NAbs on M3 will also have high levels at M9. Similarly, high levels at D36, i.e., at the highest inhibition reached two weeks after the second vaccination, are likely to result in elevated levels nine months later.

On the other hand, BMI and age are in the right/lower part of the graph with positive values with respect to the first principal component (loadings with respect to the first component: 0.26 (age) and 0.09 (BMI)). This means that BMI and age contribute negatively to the three NAbs levels. However, the angle between these two characteristics (age or BMI) and the three NAbs vectors is close to 90°, which means that their influence should be small. As expected, the variables BMI and age were found to be related, implying that as age increases, BMI also increases.

**Figure A4:**
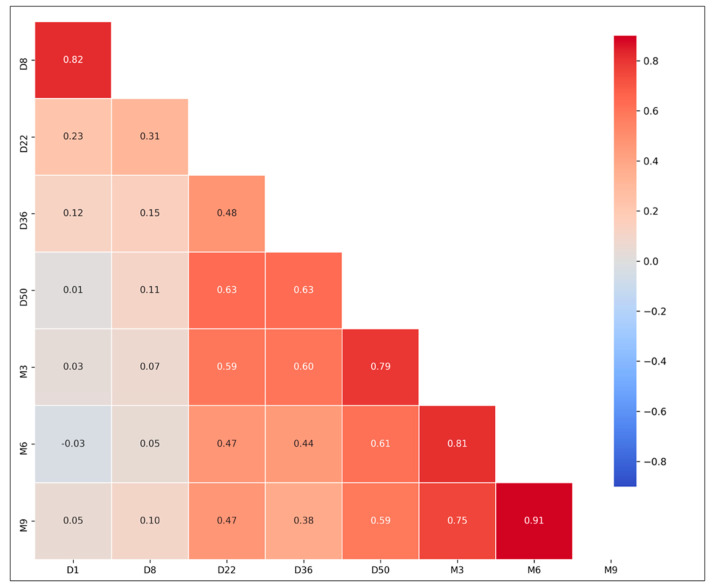
Correlation matrix showing the correlation coefficients (Spearman’s) between the numerical variables used in the analysis.

Correlation between all variables was assessed to exclude variables with strong linear relationships. Spearman’s correlation coefficients (r) are shown in Figure A4. From this analysis, NAbs values at M1 (i.e., one month after the second vaccination), M3, M6, and M9 are moderately or strongly correlated. Also, NAbs values at D1 and D8 are strongly associated with each other (Spearman’s r = 0.82). Therefore, the NAbs values at D36, M3, and M9 were included in the final PCA model.

In the same plot with PCA, the k-means cluster analysis yielded five natural groups of individuals according to their characteristics (age, BMI, sex, height, medical history, medication, etc.), with each group having a different color (Figure 2). These k-means cluster groups are consistent with the groups identified by PCA. The estimated silhouette score was 0.45, whereas the Davies-Bouldin score for the final best model was 0.57; both indices indicate adequate cluster discrimination. Several seeds were used for k-means clustering, all of which yielded nearly identical results, confirming the clustering results.

Within each group, the characteristics of some individuals differ in the sense that one characteristic (e.g., BMI) is different in a particular group than in the other clusters. In this context, the five natural groups refer to obese individuals, young individuals, individuals with typical characteristics (i.e., not elderly or obese, etc.), and participants with low NAbs at D6 or M3/M9 (Table 3).

In the first group, the distinguishing feature is mainly the low number of NAbs at D36 (mean = 69.46%) and secondly the low inhibition threshold at M3 (mean 79.84%). Of course, there is another group of subjects whose salient feature is the relatively low number of NAbs at M3 (76.26%) and M9 (mean = 41.13%). The third group of participants consists of obese subjects (mean BMI = 32.64). Young individuals with a mean age of 32.25 years form the fourth group, while the remaining participants with typical values for all five characteristics form the last group.

In addition, the topological properties of the clusters and PCA vectors can be used to determine the relationship between them (Figure 2). For example, obese subjects tend to have low NAbs, whereas young subjects or subjects with typical traits have higher inhibitory values. Subjects with “typical” traits have intermediate performance, as they fall between obese and young subjects. The highest immunological response nine months after vaccination is seen in subjects belonging to the last group, namely with BMI = 24.83 and age = 55.99 years. The lowest NAbs values at M9 (and M3) are observed in subjects who have a combination of conditions: Age between 47–50 years and BMI close to 24 (i.e., groups 1 and 2 in Table 3).

### 3.4. Factor Analysis of Mixed dData

Principal component analysis is useful for reducing the dimensionality of the data set and identifying patterns in the data. However, information from categorical variables such as sex, concomitant diseases, medications, and whether the subject has had a previous COVID19 cannot be used because PCA works only for numeric data. In addition, to investigate the possible influence of both numeric and categorical variables, a factor analysis of mixed data was performed in this study. In this case, all available information from the individuals participating in the study was analyzed. Factor analysis of mixed data was applied several times by including or excluding certain variables to find the model that explained the most variability in the data.

**Figure 3:**
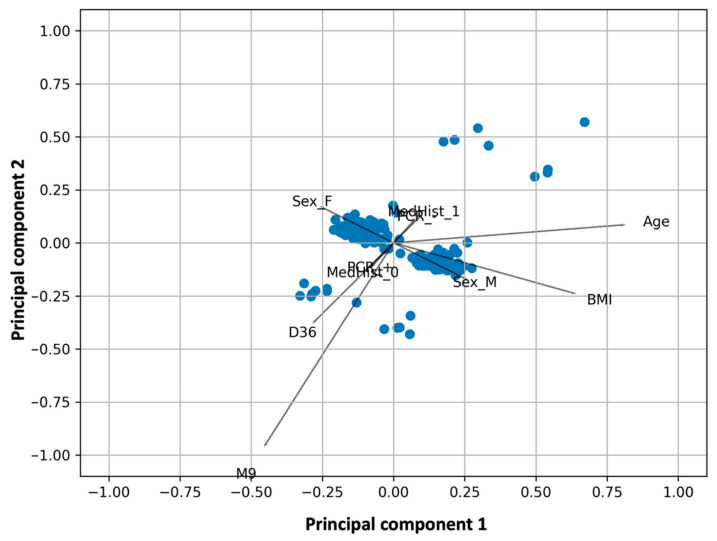
Biplot for factor analysis of mixed data. The coordinates of the two-dimensional plot refer to the “scores” of the variables in the dimensionality-reduced space (two principal components are shown in the plot). The lines represent the vectors of the variables in the 2D space. *Key*: D36, neutralizing antibody levels two weeks after second vaccination; M9, neutralizing antibody levels nine months after second vaccination; BMI, body mass index; MedHist_1, subjects with autoimmune disorders; MedHist_0, subjects without autoimmune disorders; Sex_M, men; Sex_F, women; PCR+, subjects with previous COVID-19 infection; PCR-, subjects without previous COVID19 infection.

The biplot, score, and loading plot of the final FAMD model are shown in Figure 3, while the scree plot, constructed to select the optimal number of principal components, is shown in Figure A1C. The total percentage of variability explained by the first two principal components is 68.2% (36.4% and 31.8% for the first and second components, respectively). Visual inspection of the FAMD results shows that the common aspects are consistent with PCA. Namely, age and BMI contribute negatively to NAbs values at D36 and M9. Again, the orthogonal arrangement of the vectors BMI and D36/M9 shows the small contribution of BMI to NAbs values. The angle between the “age” vector and the two NAbs vectors is obtuse, indicating that “age” certainly makes a negative contribution to neutralizing antibodies. Young people tend to have higher neutralizing antibody levels than older people. The fact that the angle (age-0-D36) is larger than (age-0-M9) means that age affects NAbs levels in D36 more than those in M9 (Figure 3). In other words, the influence of age is stronger at time points close to the vaccination day, and the role of age seems to decrease with time.

In addition, the FAMD biplot can provide further insight into the role of categorical variables. In this context, gender does not seem to affect NAbs levels at M9 (mainly), but there seems to be a weak relationship at D36. In the latter case, it appears that female subjects (sex = 2 in the graph) are associated with higher neutralizing antibody levels two weeks after the second vaccination, i.e., at D36. In the FAMD graph, it is noticeable that the performance of the two sexes is exactly opposite because their vectors are in opposite directions, i.e., their loadings are opposite with respect to both axes.

Figure 3 also shows that a previous positive PCR test (i.e., individuals infected with SARS-CoV2) has no effect on titers at M9, but PCR+ is strongly related to inhibition values at D36. As expected, earlier infection with COVID-19 leads to higher values at D36 (mainly) and nine months later (i.e., at M9).

Finally, an important finding regarding the role of comorbidities can be seen in Figure 3. Due to the limited sample size, subjects were divided into two categories: those with autoimmune diseases and those without autoimmune diseases, i.e., those who were completely healthy or suffered from other diseases such as cardiovascular disease, mental illness, etc. FAMD analysis revealed that the vector of patients with autoimmune diseases (MedHist_1) is arranged in the opposite direction to vectors D36 and M9, indicating that subjects with autoimmune diseases have lower inhibitory levels compared to those without autoimmune problems. This feature is evident in NAbs levels early after vaccination, e.g., two weeks after the second vaccination, but to a lesser extent in NAbs titers nine months after full vaccination.

### 3.5. Random Forest

**Figure 4:**
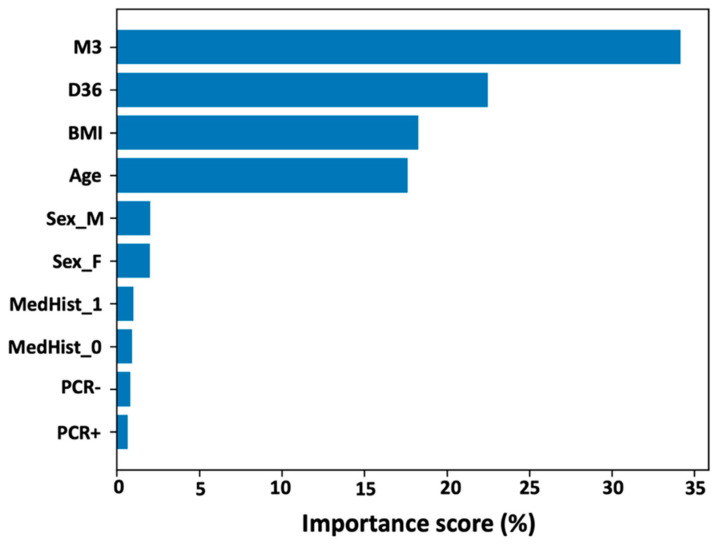
Importance scores for the feature parameters of the subjects participating in the study with regard to their contribution in predicting neutralizing antibody levels nine months after the second vaccination. *Key*: D36, neutralizing antibody levels two weeks after second vaccination; M3, neutralizing antibody levels three months after second vaccination; BMI, body mass index; MedHist_1, subjects with autoimmune disorders; MedHist_0, subjects without autoimmune disorders; Sex_M, men; Sex_F, women; PCR+, subjects with previous COVID-19 infection; PCR-, subjects without previous COVID19 infection.

To quantify the predictive factors for NAbs levels nine months after complete vaccination with BNT162b2, the random forest technique was used. Figure 4 shows the importance of the variables included in the analysis, ordered from highest to lowest contribution to M9 values. It is plausible that NAbs levels at M3 have the highest contribution (score = 34.2%), followed by inhibition titers at D36 (score = 22.5%). BMI and age, which already had a negative effect on M9 levels in the PCA and FAMD analyzes, also contribute to a lesser but important extent: 18.3% and 17.6% for BMI and age, respectively. Gender has a small effect on M9 values, as significance estimates were 2.0% for men and 2.0% for women. Medical history, i.e., whether you have an autoimmune or other disease (or are healthy), has a small effect on M9 levels (value = 1.0%). Finally, previous infection with SARS-CoV-2 before the first vaccination has a negligible effect on M9 inhibition titers. It should be mentioned that a 2:1 split (training set: test set) was used. In addition, multiple replicates of the 2:1 split were used to ensure the validity of the results.

**Figure 5:**
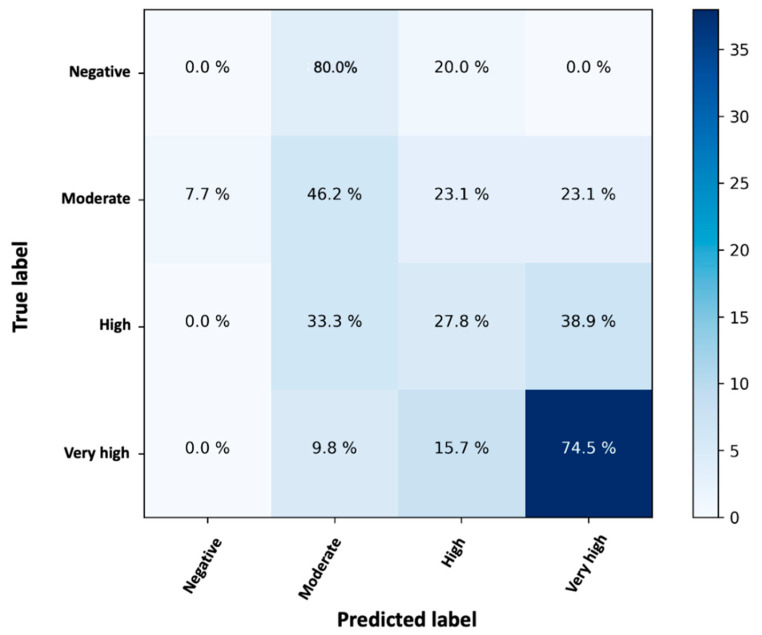
Confusion matrix of the random forest classifier to indicate the percentage of correct or incorrect predictions. The labels “negative”, “moderate”, “high”, and “very high” refer to neutralizing antibody levels (0–30%), (30–50%), (50–75%), (>75%).

To express how many of the predictions of a random forest classifier were correct and when they were incorrect (i.e., the RF classifier becomes “confused”), a confusion matrix was created (Figure 5).

The rows of the confusion matrix indicate the observed (true) labels, while the columns represent the predicted (expected) labels. The values within the matrix refer to the percentages of cases assigned to each case, as this is a normalized confusion matrix. Thus, the diagonal values indicate the percentage of cases in which the predicted label matches the observed one. The values in the other cells represent cases where the classifier misidentified an observation. The developed model RF is able to correctly classify 74.5% of very high NAbs values, 46.5% of medium values, and 27.8% of high values (Figure 5). No predictions were made for negative values because there were no subjects belonging to this category.

The prediction of very high values is accurate, but it was less accurate for “medium” and “high” values. The overall prediction accuracy of the RF classifier was 66.32%. This value could be higher if more participants were available for the “negative” group. However, since no data were available for the negative class and most of the data involved very high inhibitions, perhaps the classes were unbalanced and there was a tendency to overpredict. Either way, a percentage of 66.7% (=27.8% + 38.9%) high NAbs were predicted to be either high or very high. Similarly, a percentage of 69.3% (=46.2% + 23.1%) truly moderate cases were predicted to be moderate or high.

## 4. Discussion

**Figure 6:**
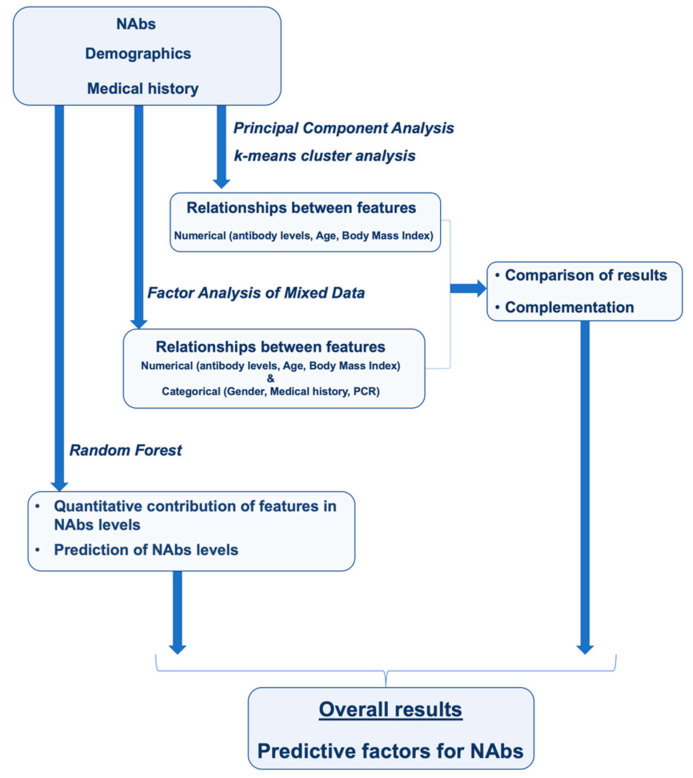
Flowchart presenting the overall route of analysis. The association between neutralizing antibody levels (NAbs) and demographics/medical history was investigated using four machine learning methods: principal component analysis, k-means cluster analysis, factor analysis of mixed data, and random forest.

The aim of this study was to use the analytical capabilities of machine learning to determine the parameters responsible for NAbs levels nine months after BNT162b2 vaccination. Data from a clinical trial of 302 subjects were analyzed to uncover correlations between subjects’ characteristics that would be difficult or impossible to detect using conventional statistical methods. Figure 6 shows the general route of analysis used in this study.

The highest levels of NAbs (median = 97.24%) were observed two weeks after the second vaccination, whereas thereafter there was a statistically significant decline up to nine months. Although the ability to neutralize SARS-CoV-2 antibodies decreased over time, it remained sufficiently high above positive thresholds; a median percent inhibition of 65.43% was observed after nine months. Other prospective studies using classical statistical methods have shown that BNT162b2 provides decreasing but significant COVID-19 protection 6 and 9 months after full vaccination [14,18,23,24,25]. Data from the field show that protective immunity against the delta version of SARS-CoV-2 decreases after several months of full immunization with BNT162b2 [16,25]. However, protection against COVID-19, particularly against severe disease, remains unchanged compared with unvaccinated individuals [16,25].

Application of PCA (Figure 2) showed the strong relationship between M9 levels and M3 levels and even earlier time points at D36, where maximum inhibition is observed. K-means clustering showed the natural grouping of subjects into five categories. Within each group, the characteristics of some subjects predominate in the sense that one characteristic is distinct from the other clusters. Young subjects, obese subjects, subjects with typical characteristics (i.e., not elderly or obese, etc.), and participants with low NAbs at D36 or M3/M9 make up the five natural groups (Figure 2). The topological characteristics of the groups as well as the PCA vectors can be used to establish their relationships. For example, obese patients have low NAbs, and accumulating data suggest that obesity may attenuate the antibody response to COVID-19 vaccination [26]. Young subjects or those with normal characteristics have higher inhibitory levels. Participants with “typical” characteristics are in the middle, between fat and young subjects. Participants in the last group, with a BMI of 24.83 and an age of 55.99 years, have the best immune response nine months after vaccination.

Factor analysis of the mixed data (Figure 3) showed that younger people have greater amounts of neutralizing antibodies than older people. Based on the observed angles between vectors, age was found to influence NAbs levels more at D36 than at M9. In other words, the influence of age is strongest near the vaccination date, and the role of age appears to decrease with time. This finding is consistent with previous studies (using typical statistical methods) indicating that older age is associated with lower humoral response after SARS-CoV-2 infection or immunization [27,28,29,30]. Compared with younger people, the elderly may have a lower capacity to produce antibodies [27]. According to one study, more than one-third of elderly vaccinated subjects lose NAb activity against the delta variant of the pathogen six months after complete immunization with BNT162b2, compared with less than 1% of younger subjects [31]. Although participants’ age has a statistically significant effect on NAbs production, as shown by NAbs levels two weeks after vaccination, this effect fades with time.

In addition, FAMD allowed examination of categorical characteristics such as gender, comorbidities, etc. Gender did not appear to affect NAbs levels at M9 (primarily), but there was a modest association with D36. In the latter situation, female subjects (sex = 2 in the graph) appeared to have higher levels of neutralizing antibodies two weeks after the second vaccination.

Regarding comorbidities, subjects were divided into two groups: those with autoimmune diseases and those without. FAMD analysis revealed that the vector of the patients with autoimmune diseases was oriented opposite to vectors D36 and M9, meaning that participants with autoimmune diseases had lower inhibitory levels than those without autoimmune diseases. These results are consistent with a previous systematic literature review study that indicated a lower antibody response after COVID-19 vaccination in individuals with rheumatic diseases compared with controls [32]. This feature is more evident in NAbs levels early after vaccination and less evident in NAbs titers nine months after full immunization.

Finally, by using the random forest technique, we were able to quantify the features predicting neutralizing antibody levels nine months after full immunization with BNT162b2. NAbs levels at M3 contribute the most, followed by levels at D36. BMI and age were found to have a small negative impact on M9, while gender had a small impact. Medical history, i.e., whether a person has an autoimmune or other disease (or is healthy), has a small effect on inhibitory levels nine months after full vaccination. Finally, prior SARS-CoV-2 infection before the first vaccination has a small effect on M9 inhibitor levels.

A limitation of this study was the small sample size, which may limit the ability to investigate specific pathophysiologic conditions. Therefore, the subgroup analyzes performed in this study should be considered exploratory. Comorbidities are known to decrease predicted immunogenicity after COVID-19 vaccination; however, this association was small between two weeks and nine months. A larger number of participants in each group is needed to find further significant differences in antibody kinetics. It is also worth noting that patients with serious comorbidities, such as cancer, were excluded from the current study, so the effects of these conditions could not be examined. The role of concomitant medications also could not be studied because the number of subjects in the study was limited compared with the large number of medications that participants were taking. Previous studies have shown that active immunosuppressive drugs can lead to poor humoral response after COVID-19 vaccination [33,34,35,36,37]. However, this aspect could not be investigated in this study. Regarding the role of gender, it should be noted that men and women received unequal sample sizes. The ratio of women to men is approximately two to one. In general, unequal sample sizes can lead to biased comparisons, but we assume that this is not a problem in our case, since the imbalance is not excessive but rather normal (33.8% versus 66.2%). The duration of the study refers to nine months after the second vaccination, almost 10 months after the first dose. Although this observation period is quite appropriate for the current time point, since vaccination programs began almost a year ago, future studies should be conducted with data for a longer period (e.g., 12 months postvaccination) to examine longer-term immune response outcomes.

## 5. Conclusions

The aim of this study was to apply four common machine learning approaches to identify the parameters responsible for NAbs levels nine months after BNT162b2 vaccination. Data from a clinical trial of 302 subjects were analyzed using principal component analysis, k-means clustering, factor analysis of mixed data, and random forest. Application of PCA revealed a strong association of M9 levels with those of M3 and even two weeks after the second vaccination, i.e., when maximal inhibition is observed. K-Means clustering showed the natural grouping of subjects into five categories, with the characteristics of some individuals predominating within each group. These five groupings refer to young subjects, obese subjects, subjects with typical characteristics, and subjects with low NAbs at D36 or M3/M9. Complementary to all these characteristics, factor analysis of the mixed data showed that young subjects had greater amounts of neutralizing antibodies compared with old subjects. Although age has a statistically significant effect on NAbs production, as shown by NAbs levels two weeks after vaccination, this effect decreases with time. In addition, participants with autoimmune diseases were found to have lower inhibitory levels than participants without autoimmune diseases. Finally, we were able to quantify the importance of person characteristics in predicting neutralizing antibody levels nine months after full vaccination using the random forest technique. NAbs levels at M3 contribute the most, followed by levels at D36, whereas BMI and age were shown to have a small negative impact on M9. Gender, previous SARS-CoV-2 infection, and medical history have also been shown to have a small effect on inhibitory levels nine months after full vaccination.

## Figures and Tables

**Table 1:** Characteristics of the study participants.

Participant Characteristic	Value (Median, Range)
Sample size	302
Gender	
Men	102 (33.8%)
Women	200 (66.2%)
Age	48 (49)
Men	49 (48)
Women	48 (46)
BMI	24.8 (27)
Men	26.8 (19.5)
Women	23.3 (27.2)

**Table 2:** Loadings for the two main principal components of the final PCA model.

Variable	Principal Component	
	1	2
**Age**	0.26	−0.60
**BMI**	0.09	−0.75
**D36**	−0.44	−0.21
**M3**	−0.57	−0.13
**M9**	−0.64	−0.09

**Table 3:** Characteristics of each group formed by k-means cluster analysis. Values refer to the mean estimates for each category.

Group	Group Label	Variable *				
		D36	M3	M9	Age	BMI
1	Low NAbs D36	69.46	79.84	69.39	47.50	23.40
2	Low NAbs M3/M9	95.09	76.26	41.13	49.51	24.30
3	Obese	97.21	90.73	58.89	50.03	32.64
4	Young Individuals	97.32	94.03	75.72	32.25	22.90
5	Typical subjects	97.10	93.51	78.65	55.99	24.83

* Values in bold indicate the “discriminating” characteristic of each cluster.

## Data Availability

The data presented in this study are available on request from the corresponding author.
